# Case Report: Hypervascular lipid-rich intrahepatic adrenocortical adenoma in the right posterior liver: a diagnostic challenge for hepatobiliary surgeons

**DOI:** 10.3389/fsurg.2026.1890393

**Published:** 2026-08-20

**Authors:** Shuqing Wu, Tong Zhou, Xingfei Li, Weihua Zhu, Jie Gao

**Affiliations:** 1 Department of Hepatobiliary Surgery, Peking University People's Hospital, Beijing, China; 2 Peking University Research Center for Diagnosis and Treatment of Liver Cancer, Beijing, China; 3 Peking University Institute of Organ Transplantation, Beijing, China

**Keywords:** adrenohepatic fusion, case report, ectopic adrenal tissue, HCC differential diagnosis, hepatectomy, hepatic adrenal rest tumor, intrahepatic adrenocortical adenoma, lipid-rich hepatic lesion

## Abstract

**Background:**

Intrahepatic adrenocortical adenoma (IAA) is a rare benign tumor that originates from ectopic adrenocortical tissue or adrenohepatic fusion. Due to its hypervascular enhancement pattern and tendency to occur in the right posterior liver, IAA may closely resemble hepatocellular carcinoma (HCC) on preoperative imaging.

**Case presentation:**

A 67-year-old woman presented with intermittent right upper quadrant discomfort for 3 months. Despite the absence of viral hepatitis and normal tumor marker levels, contrast-enhanced magnetic resonance (MR) imaging showed a well-circumscribed hypervascular lesion in liver segments VI/VII, with marked arterial phase hyperenhancement, relative portal venous phase hypoenhancement, and hypointensity during the hepatobiliary phase, initially creating an imaging appearance that raised concern for HCC. Contrast-enhanced computed tomography (CT) showed a low-attenuation lesion with a negative attenuation value on unenhanced images (-3 Hounsfield units), indicating lipid-rich content. Because malignancy could not be excluded, a laparoscopic partial hepatectomy was performed. Histopathology demonstrated an adrenocortical neoplasm composed of polygonal cells arranged in nests and cords. Immunohistochemistry was positive for steroidogenic factor-1, inhibin-alpha, and calretinin, whereas hepatocellular markers were negative. The final diagnosis was IAA. The postoperative course was uneventful, and no recurrence or metastasis was observed at 6-month follow-up.

**Conclusion:**

IAA should be included in the differential diagnosis of hypervascular hepatic lesions adjacent to the right adrenal gland, especially in patients without typical HCC risk factors or tumor marker elevation. Increased awareness of this diagnostic pitfall is crucial for facilitating multidisciplinary assessment and enabling more individualized surgical management.

## Introduction

Hepatocellular carcinoma (HCC) is a major cause of cancer-related mortality worldwide (1). In patients at risk for HCC, such as those with chronic viral hepatitis or cirrhosis, a hepatic lesion with arterial phase hyperenhancement followed by portal venous or delayed phase washout on contrast-enhanced computed tomography (CT) or magnetic resonance imaging (MRI) may permit non-invasive diagnosis (2). However, this imaging-based approach should be applied cautiously in patients who lack typical HCC risk factors, as several benign and malignant hepatic lesions can share similar enhancement patterns and may lead to diagnostic misclassification or overtreatment (3).

Intrahepatic adrenocortical adenoma (IAA) is an exceptionally rare benign tumor arising from ectopic adrenocortical tissue or adrenohepatic fusion. Related lesions have also been termed hepatic adrenal rest tumors. They typically occur in the right posterior liver adjacent to the right adrenal gland and may demonstrate hypervascular enhancement, making preoperative differentiation from HCC challenging (4). Owing to its rarity and similar imaging features, IAA is usually diagnosed only after histopathological examination of resected specimens.

We report a lipid-rich IAA in hepatic segments VI/VII that was initially suspected to represent HCC on preoperative imaging and was resected because malignancy could not be excluded. This case underscores the importance of lesion location, absence of HCC risk factors, and lipid-rich imaging features in the diagnosis of hypervascular hepatic tumors. Recognition of these indicators can help hepatobiliary surgeons and radiologists consider adrenal-origin tumors in the differential diagnosis, thereby supporting more individualized surgical planning.

## Case presentation

### Patient information and clinical findings

A 67-year-old woman presented with intermittent right upper quadrant discomfort for 3 months. Physical examination on admission had no significant abnormalities. She had no documented hypertension, hypokalemia, Cushingoid features, or other clinical manifestations suggestive of overt adrenal hormone excess. Because an adrenal-origin lesion was not considered preoperatively, a comprehensive endocrine evaluation was not performed. Therefore, subclinical hormonal secretion by the lesion could not be completely excluded. The clinical course and key diagnostic and therapeutic events are summarized in Table 1.

**Table 1:** Timeline of clinical course.

Date/period	Clinical event	Key findings
3 months before admission	Symptom onset	Intermittent right upper quadrant discomfort.
Admission	Initial evaluation	No abnormal physical findings; normal liver function; negative HBV/HCV markers; normal AFP, CEA, and CA19-9.
Preoperative imaging	MRI and CT assessment	MRI showed arterial phase hyperenhancement and relative hypoenhancement in the portal venous phase, whereas unenhanced CT demonstrated negative attenuation (−3 HU), suggesting lipid-rich content.
Preoperative assessment	Diagnostic consideration	HCC was suspected, but the absence of HCC risk factors and lipid-rich imaging features suggested an atypical lesion.
Surgery	Laparoscopic partial hepatectomy	Yellowish, firm, well-circumscribed tumor; no gross hemorrhage or necrosis.
Postoperative pathology	Final diagnosis	Adrenocortical neoplasm positive for SF-1, inhibin-alpha, and calretinin; hepatocellular markers negative; diagnosis of IAA.
Postoperative day 14	Discharge	Uneventful recovery.
6 months after surgery	Follow-up	No recurrence or metastasis.

### Laboratory findings

Laboratory investigations revealed no evidence of viral hepatitis, with negative results for hepatitis B surface antigen and hepatitis C antibody. Liver function tests remained within normal limits. Serum tumor markers, including alpha-fetoprotein (AFP), carcinoembryonic antigen (CEA), and carbohydrate antigen 19-9 (CA19-9), were also within their respective normal ranges.

### Imaging findings

**Figure 1:**
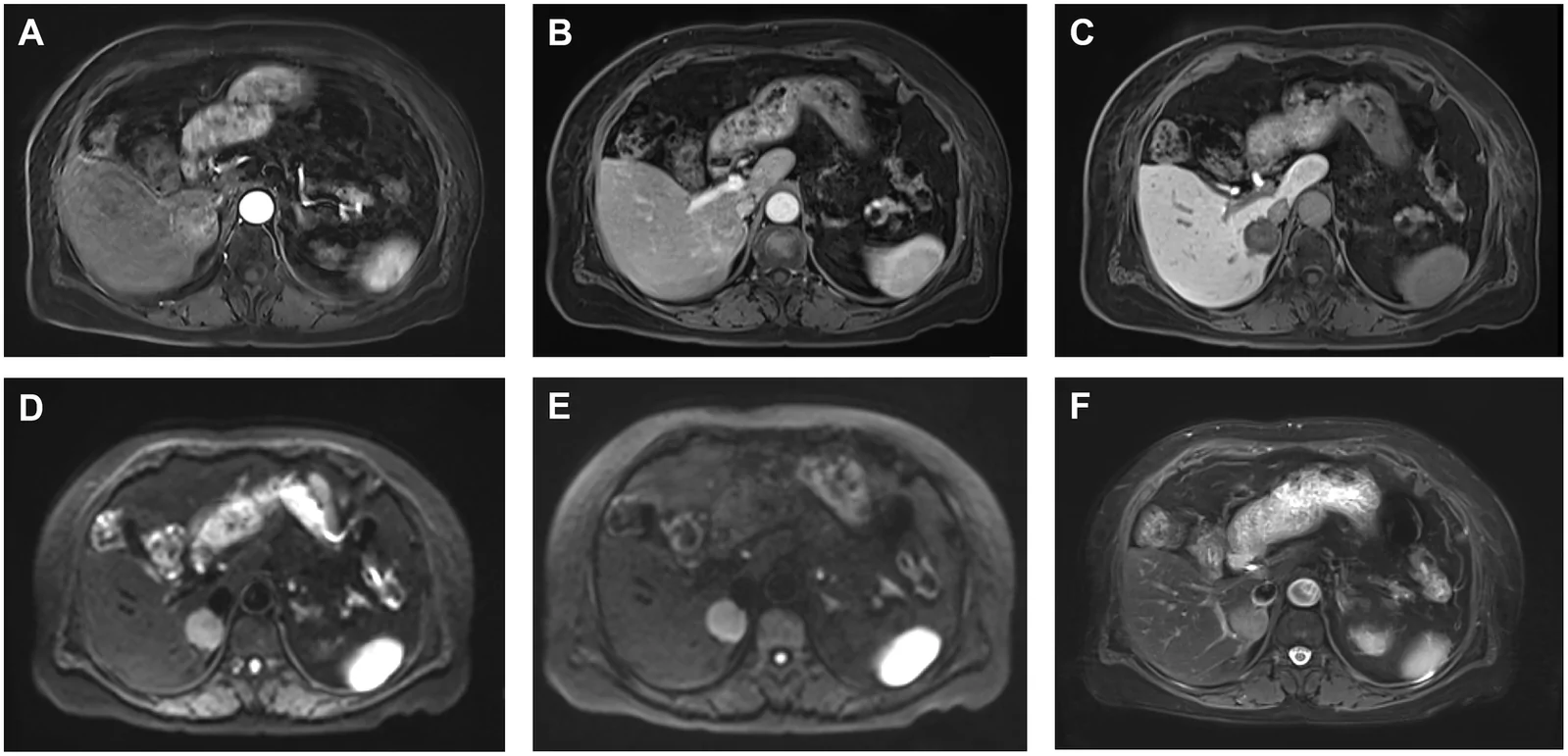
Magnetic resonance imaging findings. **(A)** Arterial phase image showing marked hyperenhancement of the lesion. **(B)** Portal venous phase image showing relative hypoenhancement compared with the surrounding liver. **(C)** Hepatobiliary phase image showing hypointensity of the lesion. **(D,E)** Diffusion-weighted images showing the lesion in the right posterior liver. **(F)** Fat-suppressed T2-weighted image showing a mildly hyperintense, well-circumscribed lesion.

Contrast-enhanced MRI revealed a well-circumscribed mass measuring approximately 2.7 × 2.0 cm located in hepatic segments VI/VII adjacent to the retrohepatic inferior vena cava and in close proximity to the right adrenal region. The lesion was slightly hypointense on T1-weighted imaging and mildly hyperintense on T2-weighted imaging. On qualitative assessment, it demonstrated marked arterial phase hyperenhancement and relative hypoenhancement compared to the surrounding hepatic parenchyma during the portal venous phase. The lesion appeared hypointense during the hepatobiliary phase. These MRI features resulted in an HCC-like imaging appearance (Figures 1A–C).

**Figure 2:**
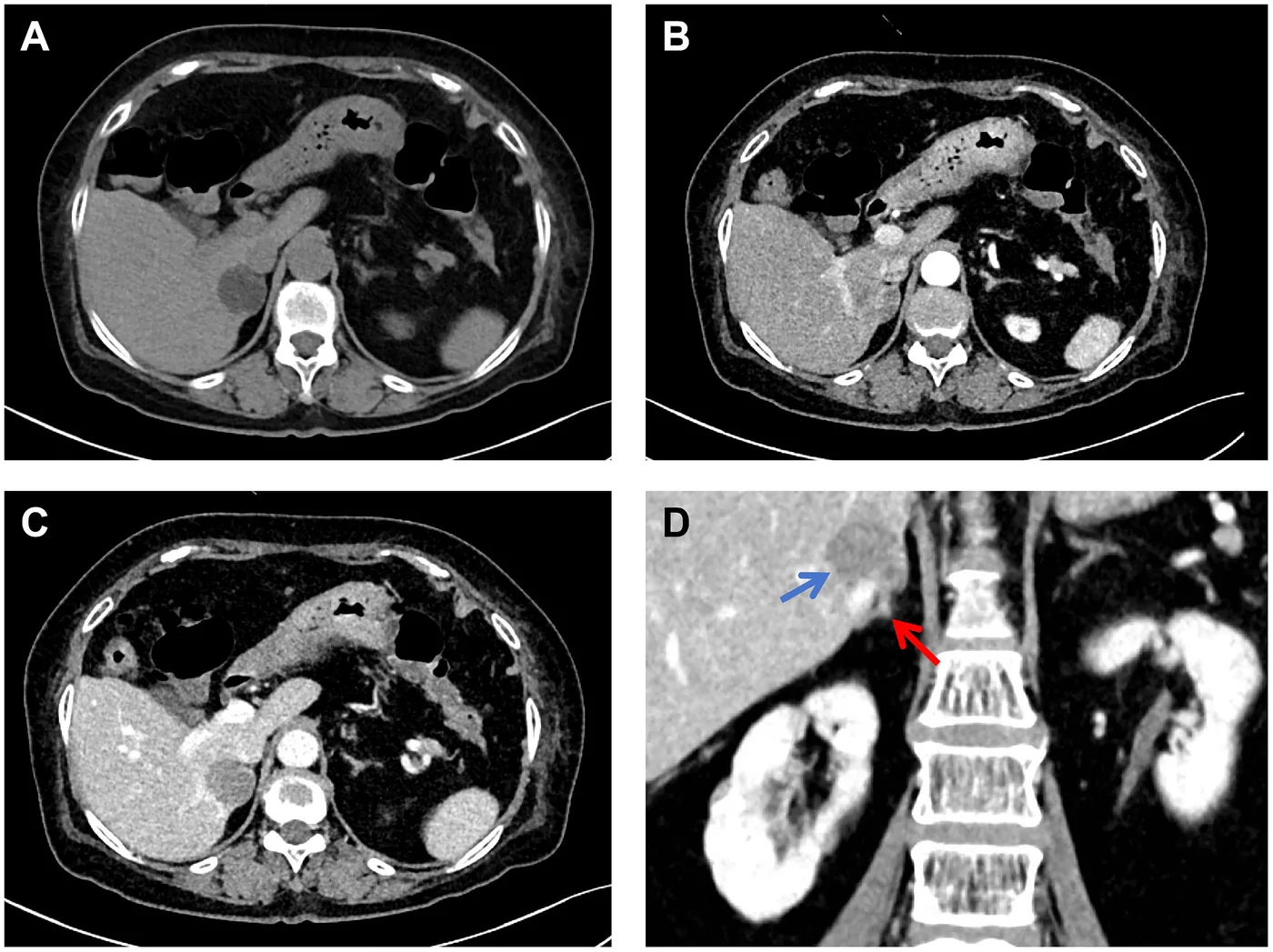
Contrast-enhanced computed tomography findings. **(A)** Unenhanced CT image showing a well-circumscribed low-attenuation lesion. **(B)** Arterial phase CT image showing marked enhancement of the lesion. **(C)** Portal venous phase CT image showing persistent enhancement of the lesion. **(D)** Coronal reconstructed image demonstrating the anatomical relationship between the tumor (blue arrow) and the right adrenal gland (red arrow).

Contrast-enhanced computed tomography (CT) demonstrated a low-density lesion in hepatic segments VI/VII with marked arterial enhancement. Attenuation values measured approximately −3 Hounsfield units (HU) on unenhanced CT, 61 HU during the arterial phase, and 78 HU during the portal venous phase (Figures 2A–C). The increase in attenuation from the arterial to the portal venous phase indicated persistent enhancement rather than typical CT washout. The negative attenuation value on unenhanced CT indicated the presence of intralesional lipid content. Coronal reconstructed CT showed that the lesion closely abutted the right adrenal region. The right adrenal gland had no obvious morphological abnormality or focal lesion on imaging. Therefore, the hypervascular appearance of the hepatic lesion, in conjunction with the location and the HCC-like MRI findings, raised concern for HCC.

### Diagnostic assessment and therapeutic intervention

The nature of the lesion remained uncertain. Although the patient had no typical risk factors for HCC, malignancy could not be ruled out because the lesion was solid and hypervascular and showed hypointensity in the hepatobiliary phase. Biopsy or imaging follow-up were not performed because the main diagnosis at that time was HCC. To preserve hepatic parenchyma, a limited partial resection of the right posterior liver was performed rather than a major hepatectomy.

**Figure 3:**
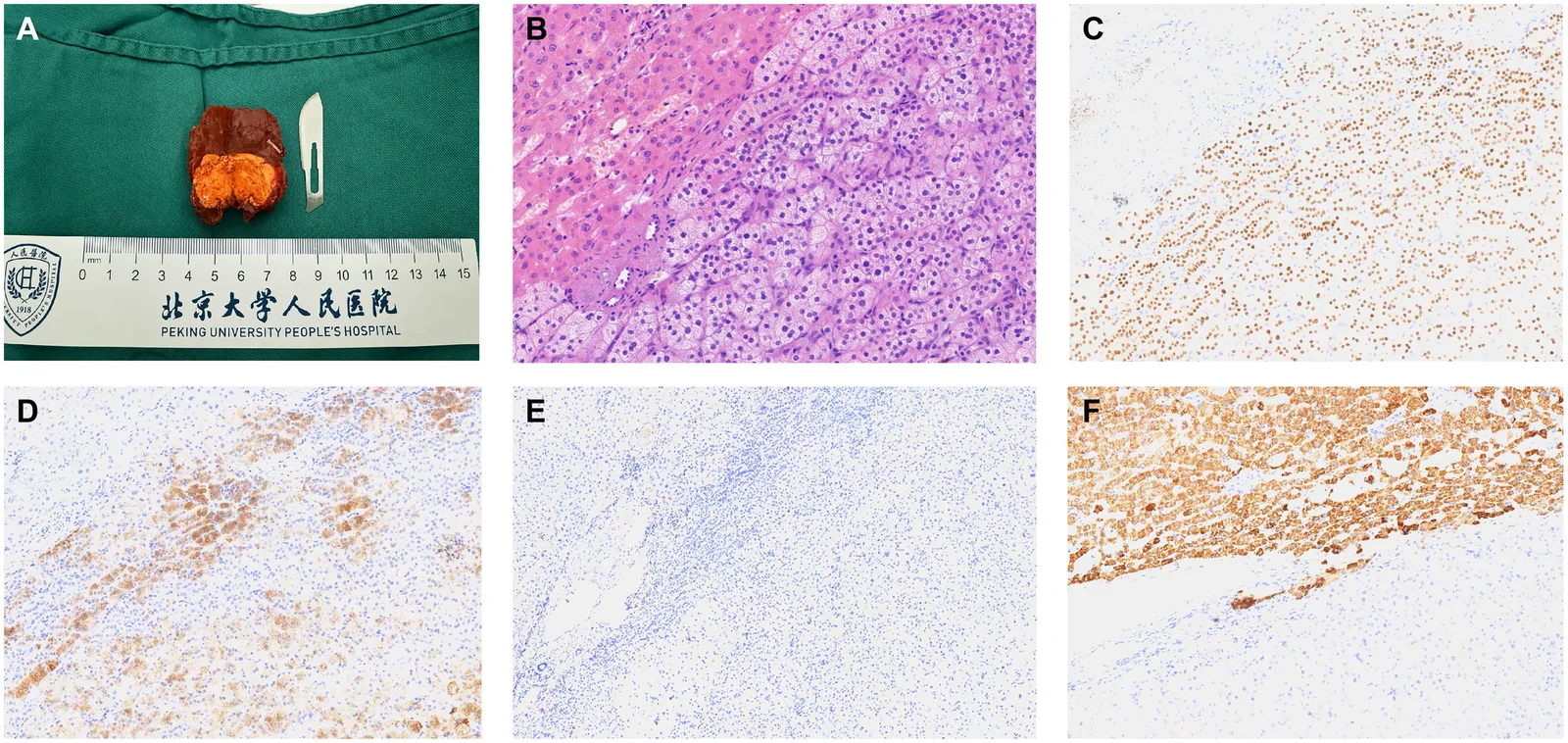
Gross and histopathological findings. **(A)** Gross specimen showing a yellowish, firm, well-circumscribed tumor without apparent hemorrhage or necrosis. **(B)** Hematoxylin and eosin staining showing a sharp interface between adjacent non-neoplastic hepatic parenchyma and the tumor, which was composed of polygonal cells arranged in nests and cords. **(C)** Immunohistochemical staining showing nuclear positivity for steroidogenic factor-1. **(D)** Immunohistochemical staining showing cytoplasmic positivity for inhibin-alpha. **(E)** Ki-67 immunostaining showing a low labeling index of approximately 1%. **(F)** Immunohistochemical staining showing no HepPar-1 expression in the tumor cells.

Intraoperatively, the tumor was yellowish, firm, and well-circumscribed, with no evidence of hemorrhage or necrosis (Figure 3A). Complete resection of the lesion was achieved, and the postoperative course was uneventful. The patient was discharged on postoperative day 14.

### Pathological findings and final diagnosis

Histopathological examination showed a neoplasm composed of polygonal cells with abundant eosinophilic to clear cytoplasm, arranged in nests and cords, morphologically consistent with adrenocortical tissue. No definitive histological features of malignancy, including tumor necrosis, marked nuclear atypia, increased mitotic activity, or vascular invasion, were observed.

Immunohistochemical staining showed positivity for steroidogenic factor-1 (SF-1), inhibin-alpha, calretinin, glutamine synthetase, and HSP70. Hepatocellular markers, such as HepPar-1, glypican-3, arginase-1, and AFP were negative. Ki-67 labeling index was approximately 1% (Figures 3C–F).

Targeted next-generation sequencing was performed, and no pathogenic or likely pathogenic variants in IGF2 were detected. This result was considered as supporting evidence instead of definitive diagnostic evidence. The distinction between adenoma from adrenocortical carcinoma was based mainly on the histological findings.

The lesion was finally diagnosed as IAA based on the characteristic morphology, adrenocortical immunophenotype, low proliferative index, and absence of malignant histological features.

### Follow-up and outcomes

Imaging showed no evidence of local recurrence or metastasis, and routine blood test results had no clinically significant abnormalities during the 6-month follow-up period. Postoperative biochemical assessment of adrenal hormone secretion was not performed. Because this interval is short for a rare adrenocortical neoplasm, continued clinical and imaging surveillance was recommended.

## Discussion

This case demonstrates a rare but clinically significant diagnostic pitfall: IAA may closely mimic HCC on preoperative imaging, particularly when presenting as a hypervascular lesion in the right posterior liver (5). The key implication of the present case is that adrenal-origin tumors should be considered when a lipid-rich hypervascular hepatic lesion is located near the right adrenal gland, especially in a patient without typical HCC risk factors or tumor marker elevation.

IAA is thought to arise from ectopic adrenocortical tissue or adrenohepatic fusion. During embryologic development, adrenal cortical tissue may become displaced or remain closely attached to adjacent hepatic parenchyma, resulting in adrenal rests within or near the liver. Although the presence of ectopic adrenal tissue and adrenohepatic fusion has been described anatomically, tumorous transformation within the liver remains exceedingly rare (6). Most reported cases have involved lesions in the right posterior liver, particularly segment VII, where the anatomical proximity to the right adrenal gland may obscure the extrahepatic or adrenal origin of the lesion (7). In the present case, the lesion was located within the right posterior liver, whereas the normally positioned right adrenal gland showed no obvious abnormality.

The terminology for these lesions is not uniform. “Hepatic adrenal rest tumor” is generally used for ectopic adrenocortical tissue or related lesions within or near the liver, whereas “intrahepatic adrenocortical adenoma” refers to a benign adrenocortical neoplasm located within the liver. Adrenohepatic fusion may also make an adrenal cortical lesion appear intrahepatic (8, 9). In our case, the lesion was located in the hepatic parenchyma, and the right adrenal gland was separately visualized without a focal mass or definite continuity with the lesion. We therefore diagnosed the lesion as IAA.

The clinical presentation of IAA is usually nonspecific (7). Patients may be asymptomatic or present with vague abdominal discomfort, and endocrine manifestations are uncommon. Therefore, imaging plays a central role in the initial evaluation. However, IAA may show marked arterial phase hyperenhancement and relative hypoenhancement or hypointensity during later phases, thereby resembling HCC. In the present case, the lesion was initially suspected to be HCC owing to its hypervascularity and hepatobiliary phase hypointensity. Nevertheless, several features argued against typical HCC, including the absence of chronic liver disease, negative viral hepatitis markers, a normal serum AFP level, and the lesion's location adjacent to the right adrenal gland.

Lipid-rich imaging features can provide a useful diagnostic clue. Many adrenal cortical tumors contain abundant intracellular lipid, which may manifest as markedly low attenuation on unenhanced CT (10). In this patient, the lesion showed a negative attenuation value on unenhanced CT (−3 HU), a finding that is uncommon for conventional HCC and should raise suspicion for an adrenal cortical lesion. When a hypervascular hepatic lesion in proximity to the right adrenal gland has lipid-rich features, comprehensive evaluation is warranted. This should include assessment of the anatomical relationship between the lesion and the adrenal gland, use of dedicated adrenal imaging protocols, endocrine evaluation, and multidisciplinary discussion to refine the preoperative diagnosis.

Histopathological examination provided definitive evidence. Immunohistochemical panels including SF-1, inhibin-alpha, and calretinin confirmed adrenocortical differentiation (11). Positivity for SF-1, inhibin-alpha, and calretinin, together with negativity for HepPar-1, glypican-3, arginase-1 strongly supported an adrenocortical rather than hepatocellular origin. Interestingly, the expression of glutamine synthetase and HSP70 formed a potential immunohistochemical pitfall, as these markers are included in diagnostic panels for hepatocellular neoplasia. However, they should not be interpreted in isolation without lineage-specific markers.

Distinguishing IAA from adrenocortical carcinoma is also important. The benign nature of the lesion was supported by its small size, low Ki-67 labeling index, and absence of overt malignant histological features. Targeted next-generation sequencing did not identify pathogenic or likely pathogenic alterations in the tested panel, including pathogenic IGF2 variants. IGF2 overexpression have been reported more frequently in adrenocortical carcinoma than in adenoma and may be used as an ancillary marker. However, most evidence for its diagnostic value is based on immunohistochemistry rather than sequence analysis (12). Therefore, negative IGF2 variants in our targeted sequencing panel was interpreted cautiously and was not considered conclusive. The distinction between benign and malignant adrenocortical neoplasms relies primarily on morphology, proliferative activity, and evidence of invasion.

From a surgical perspective, recognition of IAA should not be interpreted as a reason to avoid resection in all cases. Because IAA was not suspected preoperatively and malignancy could not be excluded, laparoscopic partial hepatectomy was performed for both diagnosis and treatment. However, the absence of chronic liver disease, normal tumor markers, negative attenuation on unenhanced CT, and proximity to the right adrenal gland should prompt further adrenal imaging and multidisciplinary assessment. Biopsy or imaging surveillance may be considered in selected cases, while limited resection remains reasonable when malignancy cannot be ruled out (8).

This report has several limitations. First, it describes a single case, and the imaging characteristics of IAA remain insufficiently defined because of the rarity of the disease. Second, the follow-up period was limited to 6 months. Although postoperative imaging and routine blood tests showed no evidence of recurrence or metastasis, longer surveillance is needed to confirm a benign clinical course. Third, neither preoperative nor postoperative biochemical assessment of adrenal hormone secretion was performed. Therefore, the functional status of the tumor could not be determined. While no overt endocrine symptoms were observed, subclinical hormone secretion remains possible. Despite these limitations, this case highlights the importance of integrating clinical background, lesion location, lipid-rich imaging features, and immunohistochemical findings when evaluating hypervascular hepatic tumors.

## Conclusion

IAA is a rare benign tumor that can radiologically masquerade as HCC. In patients without typical HCC risk factors, a hypervascular lesion in the right posterior liver adjacent to the right adrenal gland should prompt consideration of an adrenal-origin tumor, particularly when lipid-rich imaging features such as negative attenuation on unenhanced CT are present. Recognition of this diagnostic pitfall may prompt adrenal-focused imaging and endocrine evaluation, improve preoperative assessment, and facilitate individualized surgical planning. Although resection may remain appropriate when malignancy remains a concern, awareness of IAA is essential for hepatobiliary surgeons, radiologists, and pathologists in the management of hypervascular hepatic lesions.

## Patient perspective

The patient reported relief after learning that the lesion was benign and that no further treatment was required after surgery. She understood the need for regular postoperative follow-up and provided written informed consent for publication of this case report and accompanying images.

## Data Availability

The original contributions presented in the study are included in the article/Supplementary Material, further inquiries can be directed to the corresponding author.
